# Transcriptomic and proteomic insights into progressive myoclonus epilepsy type 1

**DOI:** 10.1242/dmm.052681

**Published:** 2026-05-05

**Authors:** Alina Malyutina, Carina Lund, Saara Tegelberg, Paula Hakala, Tuula A. Nyman, Anna-Elina Lehesjoki, Tarja Joensuu

**Affiliations:** ^1^ Folkhälsan Research Center, 00290 Helsinki, Finland; ^2^ Medicum, Faculty of Medicine, University of Helsinki, 00014 Helsinki, Finland; ^3^ Orion Corporation, 02200 Espoo, Finland; ^4^ Department of Immunology, Institute of Clinical Medicine, University of Oslo and Oslo University Hospital, 0372 Oslo, Norway

**Keywords:** EPM1, Cystatin B, *Cstb^−/−^* mouse, Brain, Transcriptomics, Proteomics

## Abstract

Progressive myoclonus epilepsy type 1 (EPM1) is a rare neurodegenerative disease caused by partial loss of function of cystatin B (CSTB), a cysteine protease inhibitor with known neuroprotective roles. The disease mechanisms remain largely unsolved, and no treatments are available to control the debilitating myoclonus in EPM1. We investigated the impact of CSTB loss on transcriptome and proteome in three regions of CSTB-deficient (*Cstb^−/−^*) mouse brain – the cerebellum, cerebral cortex and hippocampus – during disease progression, providing comprehensive insights into the molecular changes and disease mechanisms. We elucidated three critical pathways as potential therapeutic targets. First, significant upregulation of immune response genes indicates heightened immune activity across all brain regions. Second, consistent downregulation of the oxidative phosphorylation pathway with differential expression of mitochondrial genes implies impaired energy metabolism primarily affecting the cerebellum. Third, upregulation of genes essential for lysosomal function with simultaneous downregulation of genes encoding proteins crucial for lysosomal acidification suggests lysosomal dysfunction as an essential pathogenetic mechanism. By combining proteome with transcriptome data, we identified clusterin, apolipoprotein E, peroxiredoxin 6, cathepsin D and aldolase C as potential biomarkers for disease progression.

## Teaser

**Summary:** This study enhances understanding of progressive myoclonus epilepsy type 1 progression through detailed transcriptomic and proteomic analyses, highlighting the roles of immune response, oxidative phosphorylation and lysosomal function in disease pathogenesis.

## INTRODUCTION

Progressive myoclonus epilepsy type 1 (EPM1) [Unverricht-Lundborg disease; Online Mendelian Inheritance in Man (OMIM) 254800] is a rare neurodegenerative disorder characterized by severely disabling and treatment-resistant myoclonus and tonic-clonic epileptic seizures manifesting between 6 and 16 years of age (Kälviäinen et al., 2008; Lehesjoki and Kälviäinen, 2020). EPM1 is caused by partial loss-of-function variants in the gene encoding cystatin B (CSTB) (Pennacchio et al., 1996; Joensuu et al., 2008; Canafoglia et al., 2012), an endogenous inhibitor of lysosomal cysteine cathepsins (Stubbs et al., 1990; Turk and Bode, 1991; Čeru et al., 2010). Individuals with EPM1 have widespread degenerative changes in the brain, affecting both white and grey matter (Koskenkorva et al., 2009, 2012; Manninen et al., 2013), but exhibit no or minor cognitive decline (Lehesjoki and Kälviäinen, 2020; Äikiä et al., 2021). The mechanistic roles of CSTB in the onset and progression of EPM1 have still not been resolved. While individuals with residual CSTB expression develop EPM1, individuals with a biallelic CSTB-null genotype exhibit severe neonatal-onset encephalopathy with developmental delay, progressive cerebral and cerebellar volume loss, hypomyelination and dyskinesia (Mancini et al., 2016; O'Brien et al., 2017; Abdel-Hamid et al., 2025). Therefore, the extent of CSTB loss directly correlates with the severity of symptoms.

CSTB is ubiquitously expressed and displays both nuclear and cytoplasmic localization and partial association with lysosomal markers (Alakurtti et al., 2005). Importantly, CSTB protects neurons from an oxidative stress-responsive increase in cathepsin B expression and activity, with CSTB deficiency sensitizing cerebellar granule neurons to oxidative stress-induced death and marked susceptibility to lipid peroxidation in CSTB-deficient (*Cstb^−/−^*) mice (Lehtinen et al., 2009). Based on recent data in bone marrow-derived macrophages, genetic (trisomic) upregulation of *Cstb* reduces generation of reactive oxygen species and inflammasome activation by downregulation of mechanistic target of rapamycin (mTOR) via increased AMP-activated kinase phosphorylation (Trstenjak-Prebanda et al., 2023). Moreover, studies in cerebellar synaptosomes from symptomatic *Cstb^−/−^* mice have revealed that alterations in mitochondrial proteome and respiration correlate with early myoclonus and neurodegeneration (Gorski et al., 2023), strengthening previous implications of compromised mitochondrial function in the early pathogenesis associated with CSTB deficiency (Daura et al., 2021). Given the function of CSTB as a regulator of chromatin structure (Daura et al., 2021), these mechanisms may be at least partially mediated through transcriptional dysregulation.

The *Cstb^−/−^* mice, which exhibit complete depletion of *Cstb* mRNA and protein, show progressive myoclonus and complex motor disturbances mirroring the phenotypic features and timeline of EPM1 relatively well (Pennacchio et al., 1998; Pollari et al., 2023). The cerebellum shows progressive granule neuron loss detectable from 1 month onwards (Pennacchio et al., 1998; Tegelberg et al., 2012). Concurrently, the white matter tracts in the cerebellum degenerate, resulting in widespread white matter atrophy and a 50% volume reduction by 6 months (Tegelberg et al., 2012; Manninen et al., 2013, 2014). The cerebrum also shows cortical neuron loss from 1 month onwards and progressive atrophy from 2 months, while the hippocampus is relatively spared (Tegelberg et al., 2012; Manninen et al., 2014). By 6 months, brain atrophy is widespread (Manninen et al., 2014).

Glial activation occurs early and is widespread in the *Cstb^−/−^* mouse brain. Pre-symptomatic microglial activation is detected in the cerebellum and parts of the cerebral cortex from postnatal day (P)14, becoming more extensive throughout the brain as the disease progresses (Tegelberg et al., 2012). In the hippocampus, early phenotypic changes are accompanied by phagocytosis defects in the dentate gyrus, yet later signs of microglial activation in other hippocampal areas are relatively mild (Tegelberg et al., 2012; Sierra-Torre et al., 2020). The onset of myoclonus coincides with astrogliosis and elevated microglial proinflammatory cytokine expression in the cerebellum and cerebral cortex (Tegelberg et al., 2012; Okuneva et al., 2015). Microarray analysis of P30 *Cstb^−/−^* cerebella has revealed upregulation of immune response-associated transcripts, including markedly increased expression of the hallmark chemokine C-X-C motif chemokine ligand 3 (*Cxcl13*) (Joensuu et al., 2014). Correspondingly, *Cstb^−/−^* microglia exhibit enhanced chemokine release and increased chemotactic activity, alongside transcriptional alterations that implicate disrupted interferon signalling (Okuneva et al., 2015; Körber et al., 2016). Furthermore, pre-symptomatic P14 and early symptomatic P30 microglia demonstrate high expression and secretion of CXCL13 (Okuneva et al., 2016). Elevated levels of pro-inflammatory cytokines and chemokines have also been detected in the serum of *Cstb^−/−^* mice, along with increased brain vascularization (Okuneva et al., 2016).

To gain insight into the molecular mechanisms driving neurodegeneration associated with CSTB deficiency, we investigated the transcriptomic and proteomic changes during disease progression in the *Cstb^−/−^* mouse cerebellum, cerebral cortex and hippocampus. We observed both shared and region-specific changes, highlighting the roles of immune activation, altered oxidative phosphorylation and lysosomal function in disease progression. These findings offer valuable insights into potential biomarkers and therapeutic targets.

## RESULTS

### Transcriptional changes in the cerebellum and the cortex drive disease progression in *Cstb^−/−^* mice

**Fig. 1.**
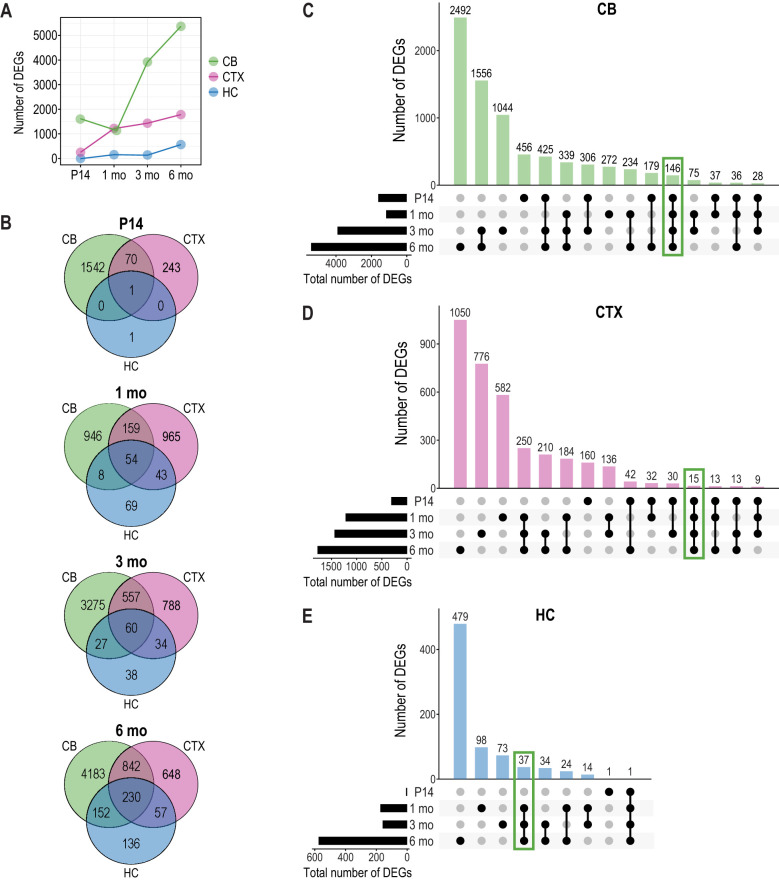
**Transcriptome profiling during disease progression in the *Cstb^−/−^* mouse brain.** (A) Effect of age on the number of significant differentially expressed genes [DEGs; adjusted *P*-value (*P*-adj<0.05)] in the cerebellum, cortex and hippocampus at postnatal day (P)14, 1, 3, and 6 months. (B) Venn diagrams showing overlap of significant DEGs (*P*-adj<0.05) between different brain regions during disease progression. (C-E) UpSet plots illustrating the number of overlapping significant DEGs between time points in cerebellum (C), cortex (D) and hippocampus (E). The top bar chart shows the number of significant DEGs for each time point combination, depicted with connected black dots in the dot matrix below. The bar chart on the left shows the total number of significant DEGs per time point. Green boxes indicate significant DEGs recurrent across all time points in the cerebellum and cortex and from 1 month onwards in hippocampus. CB, cerebellum; CTX, cortex; HC, hippocampus.

To identify molecular changes occurring during disease progression in the *Cstb^−/−^* mouse brain, we performed transcriptome profiling in three brain regions – cerebellum, cerebral cortex and hippocampus. We focused on four time points: P14, the pre-symptomatic stage with early microglial activation; 1 month, the clinical onset stage featuring myoclonic seizures and widespread microglial and astroglial activation including the cerebellum and the cerebral cortex, but not the hippocampus; 3 months, marked by progressive neuronal cell death and brain atrophy; and 6 months, marked by widespread brain atrophy. Principal component analysis of the transcriptome data revealed that segregation of the *Cstb^−/−^* genotype from wild-type (wt) controls was most evident in the cerebellum and the cortex (Fig. S1A,B), especially at P14 and 1 month, with less distinct segregation observed in the hippocampus (Fig. S1C). The differential expression analysis (DEA) revealed a progressive increase in the number of significant differentially expressed genes [DEGs; adjusted *P*-value (*P*-adj)<0.05] with age and disease progression (Fig. 1A; Table S1). The cerebellum showed the most pronounced increase, with DEGs rising from 1613 at P14 to 5407 at 6 months. In contrast, the cerebral cortex exhibited a gradual increase, from 314 at P14 to 1777 at 6 months (Fig. 1A). The hippocampus showed the fewest changes, with 575 DEGs at 6 months (Fig. 1A).

We next examined the overlap of significant DEGs (*P*-adj<0.05) between the cerebellum, cortex and hippocampus at each time point. The number of overlapping DEGs in these brain regions increased with age: one at P14, 54 at 1 month, 60 at 3 months, and 230 at 6 months (Fig. 1B). More overlap was found between the cerebellum and the cortex, with 842 overlapping DEGs at 6 months. However, the majority of cerebellar DEGs remained unique at the four time points.

To visualize the extent to which the significant DEGs within each brain region showed differential expression at multiple time points, we created UpSet plots (Lex et al., 2014) that summarize the number of DEGs overlapping between different time points (Fig. 1C-E). In the cerebellum, we identified 146 significant DEGs present at all four time points (Fig. 1C; Fig. S2A). Between clinical onset at 1 month and the stage of widespread brain atrophy at 6 months, a total of 339 significantly DEGs were shared (Fig. 1C). The cerebral cortex exhibited 15 significant DEGs that showed consistent differential expression at all four time points investigated (Fig. 1D; Fig. S2B). From symptom onset onwards, 250 genes remained differentially expressed (Fig. 1D). In the hippocampus, from 1 month onwards, 37 significant DEGs showed consistent differential expression (Fig. 1E; Fig. S2C). The one DEG detected at all time points in the hippocampus was *Cstb.*

To identify transcriptional changes common to the brain regions, we analysed genes that were differentially expressed in multiple regions over time. Twenty-seven genes (*Cstb* included) were found to be differentially expressed in all three brain regions, with 26 genes remaining consistently upregulated in at least three time points during disease progression (Fig. S3). Among these 26 genes, ten are linked to reactive astrocytes (Zamanian et al., 2012; Hasel et al., 2021) and ten to disease-associated microglia (Keren-Shaul et al., 2017).

### Pathway analysis implies alterations in immune response, oxidative phosphorylation, and lysosomal function in the *Cstb^−/−^* mouse brain

**Fig. 2.**
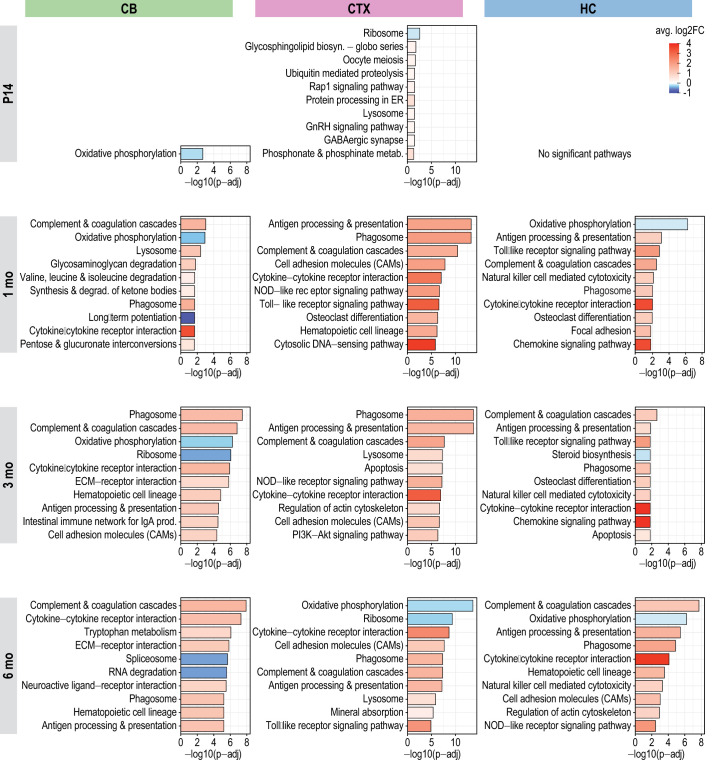
**Gene set enrichment analyses (GSEA) for Kyoto Encyclopedia of Genes and Genomes (KEGG) pathways across disease progression in the *Cstb^−/−^* mouse brain.** Top ten most significant KEGG pathways in the cerebellum, cortex and hippocampus at different time points during disease progression, based on their *P*-adj. The *x*-axis represents statistical significance as −log10(*P*-adj). The colour scale indicates the average directional log2-fold change (log2FC) with blue representing downregulation and red representing upregulation of genes within each pathway in the differential expression analysis.

To gain a comprehensive understanding of the potentially involved pathways, we performed ensemble of gene set enrichment analyses (EGSEA) for the Kyoto Encyclopedia of Genes and Genomes (KEGG) (Table S2) pathways from the *Cstb^−/−^* mouse transcriptome data. We first examined the top ten most significant KEGG pathways [−log10(*P*-adj)] for each brain region and time point separately (Fig. 2). At P14, ‘oxidative phosphorylation’ was the sole pathway significantly enriched in the *Cstb^−/−^* cerebellum, remaining among the most enriched pathways at 1 and 3 months. From 1 to 6 months, significantly enriched pathways in the cerebellum included ‘complement and coagulation cascades’, ‘phagosome’ and ‘cytokine-cytokine receptor interaction’. These pathways also showed enrichment in the cerebral cortex and the hippocampus, with ‘ribosome’ and ‘lysosome’ emerging as additional enriched pathways, especially in the cortex, as the disease progressed (Fig. 2).

**Fig. 3.**
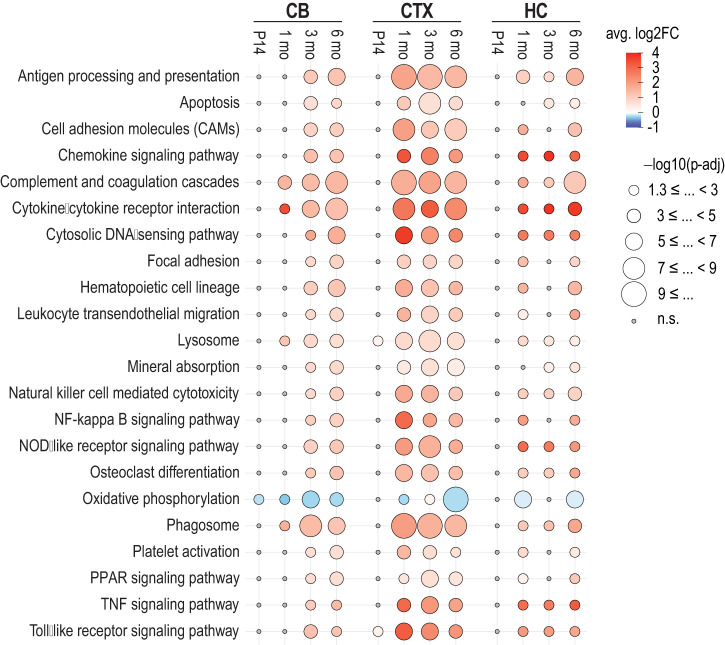
**Shared KEGG pathway enrichments across *Cstb^−/−^* mouse brain regions.** Pathway enrichment dot plot highlighting the most significantly enriched KEGG pathways across the three *Cstb^−/−^* mouse brain regions. Circle size indicates statistical significance as −log10(*P*-adj), while colour intensity denotes the average directional log2FC, with blue representing downregulation and red representing upregulation. n.s., non-significant with *P*-adj>0.05.

Next, we analysed shared enriched KEGG pathways across the three *Cstb^−/−^* mouse brain regions and four time points to identify potential pathway overlaps. We identified 22 pathways that were significantly enriched at a minimum of two time points across all brain regions (Fig. 3; Table S3). The ‘lysosome’ pathway was significantly enriched and consistently upregulated in all brain regions and time points, except for P14 in the cerebellum and hippocampus, while the phagosome pathway was upregulated in all brain regions and time points, with the exception of P14 across all brain regions. ‘Oxidative phosphorylation’ was significantly enriched and consistently downregulated only in the cerebellum at all time points, while in the cortex and hippocampus, it was significantly downregulated at 1 and 6 months. Notably, ten pathways were linked to immune system responses. In addition, there were 13 pathways unique to the cerebellum, mainly related to energy metabolism (Fig. S4A), and three unique to the cortex (Fig. S4B).

### DEGs suggest a robust immune system response in the *Cstb^−/−^* mouse brain

**Fig. 4.**
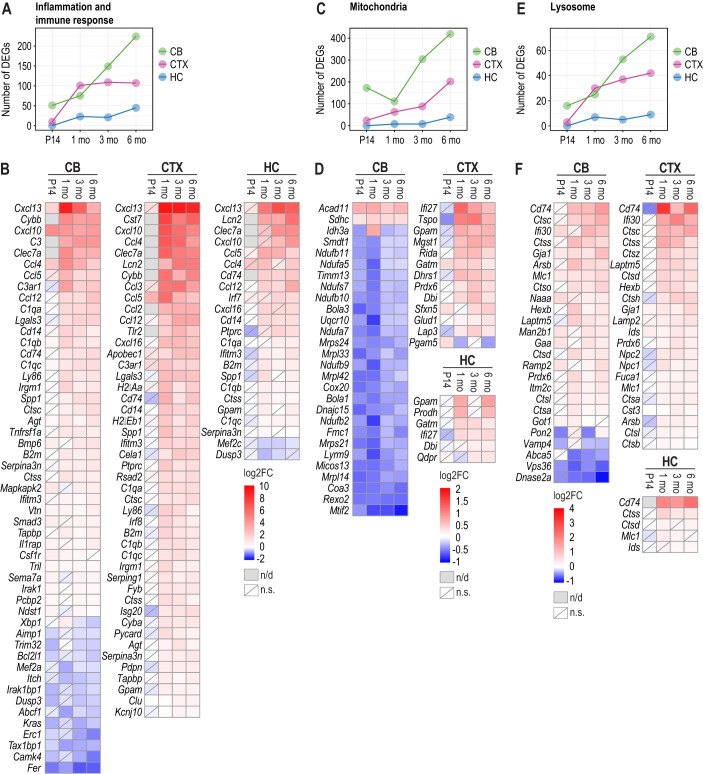
**Differentially expressed genes linked to significantly enriched Gene Ontology (GO) terms related to inflammation and immune response, mitochondria, and lysosomes during disease progression in the *Cstb^−/−^* mouse brain.** (A) The number of significant DEGs linked to GO gene sets associated with inflammation and immune response-associated pathways in the cerebellum, cortex and hippocampus at P14, 1, 3 and 6 months. (B) Heatmaps illustrating the top inflammation and immune response-associated DEGs that were significant in at least three time points in the cerebellum and cortex and at least two time points in the hippocampus. (C) The number of significant mitochondrial DEGs based on MitoCarta 3.0 in cerebellum, cortex and hippocampus at P14, 1, 3 and 6 months. (D) Heatmaps illustrating the top mitochondrial DEGs that were significant at all time points in the cerebellum, at least three time points in the cortex, and at least two time points in the hippocampus. (E) The number of significant DEGs linked to GO gene sets associated with lysosome pathway in cerebellum, cortex and hippocampus at P14, 1, 3 and 6 months. (F) Heatmaps illustrating the top lysosome-associated DEGs that were significant in at least three time points in the cerebellum and cortex and at least two time points in the hippocampus. n/d, not determined; n.s., non-significant with *P*-adj>0.05.

Next, we analysed DEGs linked to Gene Ontology (GO) gene sets related to immune system and inflammation response pathways (Table S4) in the *Cstb^−/−^* brain (Table S5). In the cerebellum, the number of these genes increased progressively, especially from 1 month onwards (Fig. 4A). In contrast, the cortex displayed a marked increase in the number of inflammation-associated DEGs only between P14 and 1 month, after which their number remained relatively stable. The hippocampus showed a modest increase in the number of inflammation-associated DEGs during disease progression. Among the inflammatory pathways, genes encoding pro-inflammatory CC cytokines (*Ccl4*, *Ccl5*), interferon-inducible pro-inflammatory cytokines (*Cxcl10* and *Cxcl13*), and markers for disease-associated microglia, including *Clec7a* and *CD74* (Potru and Spittau, 2023; Yang et al., 2025), were upregulated at a minimum of two time points in all three brain regions (Fig. 4B). In the cortex and the hippocampus, progressive upregulation of lipocalin 2 (*Lcn2*), a marker for reactive astrocytes (Zamanian et al., 2012), highlighted increasing astroglial reactivity from 1 month onwards.

### Mitochondrial gene expression is reduced with age, particularly in the cerebellum of *Cstb^−/−^* mice

According to the EGSEA KEGG pathway analyses, ‘oxidative phosphorylation’ was the only pathway that exhibited consistent downregulation with significant enrichment in the cerebellum at all time points (Fig. 3; Table S2). EGSEA analysis for GO pathways (Table S6) indicated significant downregulation of the mitochondrial respiratory electron transport chain pathway as early as P14 in the cerebellum. In contrast, in the cortex, it was significantly upregulated at 1 and 3 months, followed by downregulation at 6 months (Table S6). To gain a deeper insight into the differentially expressed mitochondrial genes, we used the MitoCarta 3.0 database (Calvo et al., 2016) for genes encoding proteins with known mitochondrial localization. Differential expression of the MitoCarta-annotated genes was detected in all brain regions, with the highest number observed in the cerebellum (Fig. 4C; Table S7). In the cerebellum, the number of differentially expressed mitochondrial genes was already high at P14 and increased markedly at 3 and 6 months (Fig. 4C). Several of these genes were consistently downregulated, including multiple NADH:ubiquinone oxidoreductase (complex I) subunit genes (*Ndufb11*, *Ndufa5*, *Ndufb10*, *Ndufa7*, *Ndufb9*, *Ndufb2*), mitochondrial ribosome genes (*Mrps24*, *Mrpl33*, *Mrpl42*, *Mrps21*, *Mrpl14*) and the translation factor *Mtif2*. Also, Fe-S cluster genes *Bola1* and *Bola3*, complex IV assembly factors *Coa3* and *Cox20*, complex V assembly factor *Fmc1*, and CIII subunit *Uqcr10* were downregulated, whereas acyl-CoA dehydrogenase (*Acad11*) and succinate dehydrogenase complex subunit C (*Sdhc*) were consistently upregulated (Fig. 4D). Mitochondrial genes consistently upregulated from 1 to 6 months in the cortex included *Ifi27*, *Tspo*, *Gpam* and *Gatm* (Fig. 4D). Notably, *Ifi27* and *Gatm* were also differentially expressed in the hippocampus from 1 month onwards (Fig. 4D).

### Gene expression alterations suggest disrupted lysosomal homeostasis in the *Cstb^−/−^* mouse brain

The ‘lysosome’ pathway was significantly enriched and upregulated in the EGSEA KEGG pathway analysis of the *Cstb^−/−^* brain in all brain regions and time points, except at P14 in the cerebellum and the hippocampus (Fig. 3; Table S2). Because CSTB is an inhibitor of lysosomal cathepsins and is reported to partially associate with lysosomes (Alakurtti et al., 2005), we analysed DEGs associated with the GO lysosome gene set (Table S8) in the *Cstb^−/−^* brain (Table S9). The number of lysosomal DEGs increased progressively in the cerebellum and cortex during disease progression (Fig. 4E). Several cathepsins and lysosomal hydrolases – including *Ctsc*, *Ctss*, *Ctsl*, *Ctsa*, *Arsb* and *Hexb* – were consistently upregulated at least at three time points in the cerebellum and cortex (Fig. 4F). Among downregulated genes, *Vps36*, encoding a component of the ESCRT-II complex involved in multivesicular body (MVB) formation and sorting of endolysosomal cargo proteins into MVBs (Babst et al., 2002; Wang et al., 2017), and *Dnase2a*, encoding a lysosomal DNA hydrolase (Lan et al., 2014), were consistently downregulated in the cerebellum from P14 onwards. Additionally, *Abca5*, encoding a membrane-associated ATP-binding cassette transporter involved in cholesterol transport, was downregulated in the cerebellum. Only a few significant lysosomal DEGs were observed in the hippocampus, with *Cd74* and *Ctss* being consistently upregulated from 1 month onwards, as in the cerebellum and cortex (Fig. 4F).

**Fig. 5.**
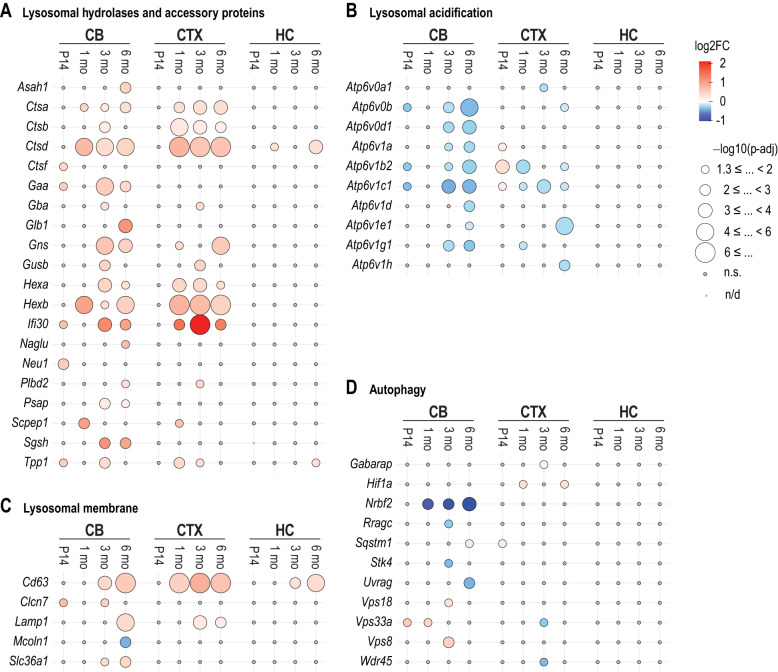
**Differential expression of Coordinated Lysosomal Expression and Regulation (CLEAR) network genes with known roles in lysosomal function in the *Cstb^−/−^* mouse brain.** (A-D) Dot plots of DEGs from the CLEAR network, which are direct targets of transcription factor EB (TFEB), in the cerebellum, cortex, and hippocampus at P14, 1, 3, and 6 months. DEGs encoding proteins that function as lysosomal hydrolases and accessory proteins (A); in lysosomal acidification (B); as lysosomal membrane proteins (C); in autophagy (D). Circle size indicates statistical significance as −log10(*P*-adj), while colour intensity denotes the average directional log2FC, with blue representing downregulation and red representing upregulation. n.s., non-significant with *P*-adj>0.05.

Lysosome biogenesis is regulated by the transcription factor EB (TFEB), which controls the expression of Coordinated Lysosomal Expression and Regulation (CLEAR) network genes (Palmieri et al., 2011; Settembre et al., 2011). Given the enrichment of lysosomal genes in KEGG pathway analysis in the *Cstb^−/−^* brain (Fig. 3 and Fig. 4E,F), we investigated whether these genes were members of the CLEAR network by identifying direct targets of TFEB with known roles in lysosomal function. We identified several differentially expressed TFEB targets, particularly in the cerebellum and cortex (Fig. 5A-D). The most marked changes involved genes encoding lysosomal hydrolases (Fig. 5A) and the lysosomal vacuolar (H^+^) ATPase (V-ATPase) (Fig. 5B). Genes encoding lysosomal hydrolases, such as cathepsins *Ctsa*, *Ctsb* and *Ctsd*, as well as metabolic enzymes *Hexa* and *Hexb*, were upregulated in the cerebellum and cortex (Fig. 5A). The lysosomal V-ATPase hydrolyses ATP to fuel transport of H^+^ into the lumen of endolysosomal membranes, maintaining the acidic luminal pH required for hydrolase activity, transmembrane protein function and lysosomal homeostasis (Sun-Wada et al., 2004). At 3 and 6 months, several genes encoding both the V1 subunit (containing catalytic sites for ATP hydrolysis) and the V0 subunit (responsible for proton translocation across membranes) of the ATPase complex were downregulated, particularly in the cerebellum. Among genes encoding lysosomal membrane proteins, the exosome, late endosome and lysosome-associated tetraspanin (*Cd63*) (Pols and Klumperman, 2009; Ai et al., 2024) was upregulated in all three brain regions (Fig. 5C). Among the autophagy-associated genes, *Nrbf2*, a nuclear receptor-binding factor involved in the regulation of autophagy, memory and long-term potentiation in mice (Lu et al., 2014; Lachance et al., 2019), was consistently downregulated in the cerebellum from 1 month onwards (Fig. 5D).

### The cerebellum shows the most pronounced changes in protein abundance

**Fig. 6.**
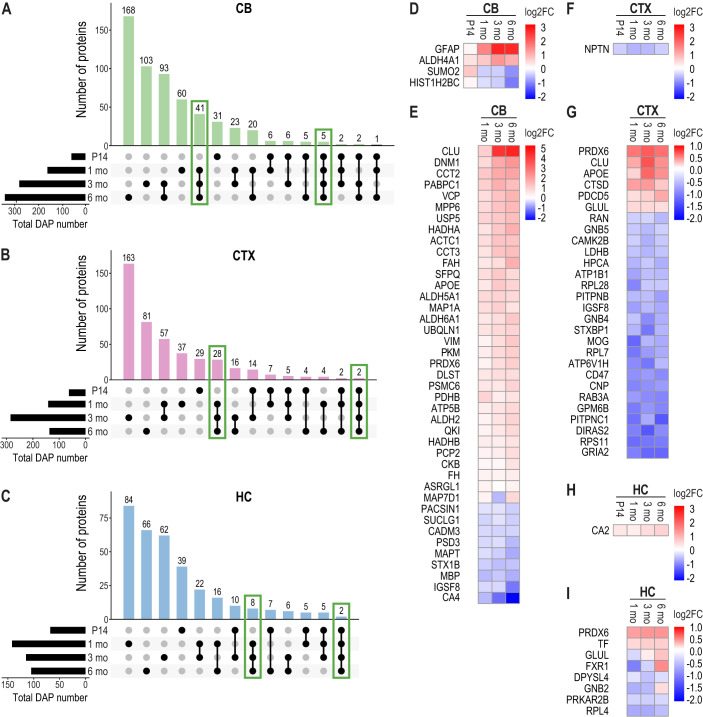
**Differential protein abundance in the *Cstb^−/−^* mouse brain.** (A-C) UpSet plots illustrating the number of overlapping differentially abundant proteins (DAPs) during disease progression in cerebellum (A), cortex (B) and hippocampus (C). The bar chart at the top shows the number of DAPs for each time point combination, depicted with connected black dots in the dot matrix below. The bar chart on the left shows the total number of DAPs. The analysis includes proteins with a *P*-value<0.05 before multiple testing adjustment. Green boxes indicate significant DAPs recurrent across all time points and from 1 month onwards within each brain region. (D-I) Heatmaps of DAPs (*P*-value<0.05) showing overlap in cerebellum from P14 to 6 months (CSTB excluded from the heatmap) (D); cerebellum from 1 to 6 months (E); cortex from P14 to 6 months (CSTB excluded from the heatmap) (F); cortex from 1 to 6 months (G); hippocampus from P14 to 6 months (CSTB excluded from the heatmap) (H); hippocampus from 1 to 6 months (I).

In addition to transcriptome analysis, we performed label-free, quantitative proteome analysis to identify differentially abundant proteins (DAPs) during disease progression across all three brain regions in *Cstb^−/−^* mice. Using the same statistical analysis methods as for the transcriptome, we discovered a relatively low number of DAPs especially at P14 and 1 month time points. Therefore, to increase the depth of the protein data, we also examined proteins exhibiting *P*<0.05 before adjustment, thereby expanding the number of proteins considered (Table S10). We examined changes in protein abundance and overlap between the three brain regions in *Cstb^−/−^* mice (Fig. 6A-C). As with the transcriptome data, the cerebellum exhibited the highest number of differentially abundant proteins (566 in total). In the cerebellum, the abundances of five proteins were altered at all four time points (Fig. 6A). Histone H2B type 1-C/E/G (HIST1H2BC) and small ubiquitin-related modifier 2 (SUMO2), both involved in post-translational modifications, showed decreased abundance (Fig. 6D), whereas the abundance of glial fibrillary acidic protein (GFAP) was decreased at P14 but then increased as disease progressed (Fig. 6D). From 1 month onwards, the abundances of 41 proteins were altered (Fig. 6A), including a significant increase in clusterin (CLU) (Fig. 6E).

In the cortex, only two proteins exhibited differential abundance from P14 to 6 months (Fig. 6B). In addition to CSTB, neuronal cell membrane protein neuroplastin (NPTN) showed decreased abundance (Fig. 6F). Among 28 differentially abundant proteins from 1 to 6 months (Fig. 6B), most showed decreased abundance. Six proteins – including cathepsin D (CTSD), glutamate-ammonia ligase (GLUL), programmed cell death protein 5 (PDCD5), peroxiredoxin (PRDX6), CLU and apolipoprotein E (APOE) – showed increased abundance (Fig. 6G).

In the hippocampus, carbonic anhydrase (CA2) showed a consistent decrease in abundance from P14 to 6 months (Fig. 6H). Additionally, eight proteins exhibited changes between 1 and 6 months (Fig. 6C), including increased abundance of PRDX6 (Fig. 6I).

### Combined transcriptome and proteome analysis reveals alterations in synaptic and lysosomal pathways in the brain of *Cstb^−/−^* mice

**Fig. 7.**
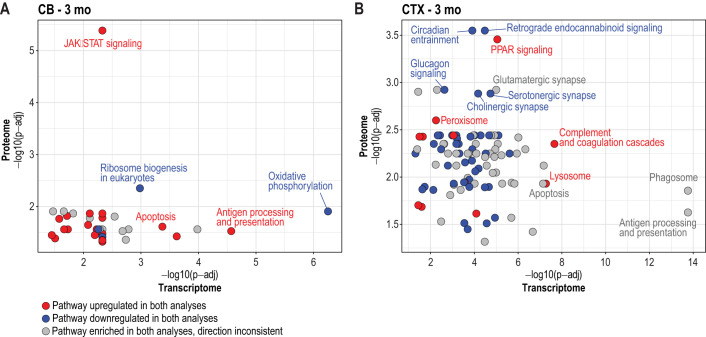
**EGSEA pathway analysis of the proteome and transcriptome in the *Cstb^−/−^* mouse brain highlights the overlap between the two datasets.** (A,B) Multiple variable plots depicting overlap of KEGG signalling and metabolic pathways between proteome and transcriptome at the 3-month time point in cerebellum (A) and cortex (B). The *y*-axis represents the proteome and the *x*-axis represents the transcriptome, with adjusted *P*-values shown on a −log10 scale. Red dots indicate significant upregulation (positive average log2FC), and blue dots significant downregulation (negative average log2FC) in both datasets. Grey dots indicate significantly enriched pathways with inconsistent direction of expression.

We next performed EGSEA pathway analysis on the protein data (Table S11), following the same approach used for the transcriptome, and investigated the overlap within these two datasets. At P14 and 1 month, only a few KEGG pathways overlapped between the transcriptome and proteome (Table S12), likely due to the relatively low number of significant DAPs in the proteome data (Table S10). At P14, the ‘ribosome’ pathway in the cortex was the only pathway significantly downregulated in both the transcriptome and proteome (Table S11). At 3 months, we identified 38 overlapping KEGG signalling and metabolic pathways in the cerebellum and 99 in the cortex (Fig. 7A,B; Table S12). In the hippocampus at 3 months, the upregulated chemokine signalling pathway was the only significantly enriched pathway. Further, at 6 months, we detected 86 overlapping pathways in the cerebellum, whereas none were found in the cortex and only two in the hippocampus (Table S12).

In the cerebellum of *Cstb^−/−^* mice at 3 months, both transcriptome and proteome analyses showed consistent upregulation of pathways such as ‘JAK-STAT’, ‘apoptosis’ and ‘antigen processing and presentation’ (Fig. 7A; Table S12). In contrast, ‘ribosome biogenesis’ and ‘oxidative phosphorylation’ were consistently downregulated in both datasets. At 3 months, the cortex showed 43 downregulated and 11 upregulated pathways, consistently observed in both the transcriptome and proteome datasets (Table S12). Downregulated pathways included, for example, ‘cholinergic synapse’, ‘serotonergic synapse’, ‘retrograde endocannabinoid signalling’, ‘glucagon signalling’ and ‘circadian entrainment’ (Fig. 7B; Table S12). A detailed analysis of the genes and proteins in the glutamatergic, serotonergic and cholinergic pathways in the cortex revealed a decreased abundance of guanine nucleotide-binding proteins (G-proteins) beta 1, 2, 4 and 5 (GNB1, GNB2, GNB4, GNB5) (Fig. S5A-D), while in the transcriptome, *Gnb1* was upregulated and *Gnb5* was downregulated. Upregulated pathways in the cortex included, for example, the ‘PPAR signalling pathway’, ‘peroxisome’, ‘lysosome’ and ‘complement and coagulation cascade’ pathways (Fig. 7B; Table S12). The ‘lysosome’ pathway was significantly upregulated at 3 months in both transcriptome and proteome EGSEA analysis, although only a few lysosomal proteins showed significant changes in abundance, including a decrease in the V-ATPase V1 subunit H (ATP6V1H) and an increase in CTSD, prosaposin (PSAP) and tripeptidyl peptidase 1 (TPP1) (Fig. S5D).

### Proteomics reveals consistently increased abundance of CLU, APOE, PRDX6, CTSD and ALDOC in the brain of *Cstb^−/−^* mice

**Fig. 8.**
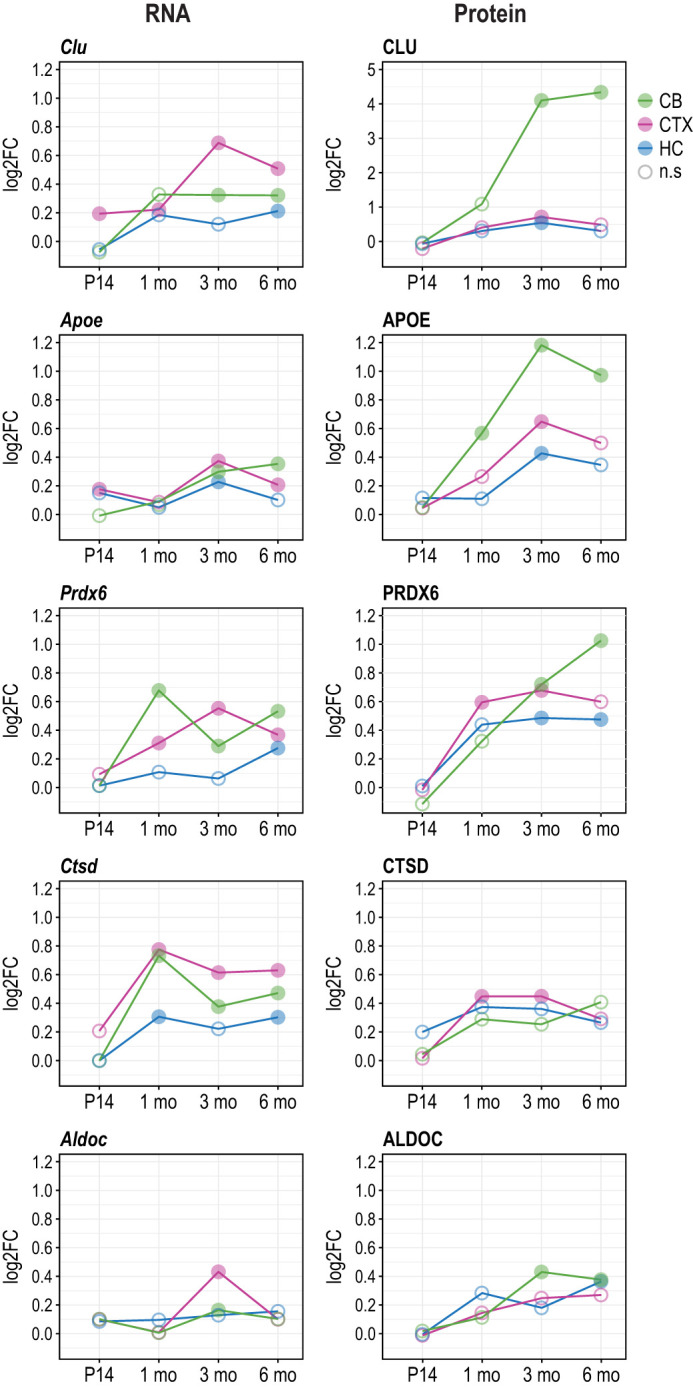
**Five proteins exhibiting increased abundance in the *Cstb^−/−^* mouse brain during disease progression, showing concordant changes at the mRNA level.** Line charts depicting differential mRNA expression (left column) and differential protein abundance (right column) in cerebellum, cortex and hippocampus at different time points of disease progression. The *y*-axis represents log2FC. Filled circles indicate significant values (*P*-adj<0.05), while unfilled circles indicate non-significant values (*P*-adj>0.05). n.s, non-significant.

Finally, we compared the DAPs to the DEGs and examined changes in more detail. We identified five proteins – CLU, APOE, PRDX6, CTSD and ALDOC – that showed increased abundance in all three brain regions during disease progression and a similar expression pattern at the mRNA level (Fig. 8; Table S13). The apolipoprotein CLU showed the highest increase in abundance, particularly in the cerebellum, with log2-fold change (log2FC) values of 4.1 at 3 months and 4.3 at 6 months. APOE exhibited steadily increasing protein levels in the cerebellum from 1 month onwards, with significantly increased abundance also observed in the cortex and hippocampus by 3 months. Similarly, PRDX6, a lysosome-associated enzyme, showed increasing abundance during disease progression. The lysosomal hydrolase CTSD and the glycolytic enzyme fructose-bisphosphate ALDOC showed modestly increased abundance, with significant changes detected only in the cortex at 1 and 3 months for CTSD and in the cerebellum at 3 and 6 months for ALDOC.

## DISCUSSION

The current study provides a comprehensive view of transcriptomic and proteomic changes in the brain of the *Cstb^−/−^* mouse model of EPM1, caused by partial loss-of-function variants that reduce, but do not abolish, CSTB expression. In contrast, complete CSTB deficiency results in a severe neurodevelopmental phenotype that is clearly distinct from EPM1, manifesting as developmental delay, progressive cerebral and cerebellar volume loss, hypomyelination and dyskinesia (Mancini et al., 2016; O'Brien et al., 2017; Abdel-Hamid et al., 2025). Although the *Cstb^−/−^* mouse does not express *Cstb* mRNA and protein, its clinical presentation and brain histopathology closely mirror the disease manifestations observed in EPM1. Importantly, detailed phenotypic characterization of the *Cstb^−/−^* mouse, including the use of novel technology for improved symptom quantification, has shown that the motor deficits in *Cstb^−/−^* mice resemble the human EPM1 phenotype more than initially described (Pollari et al., 2023). We chose three brain regions as they present different disease-associated pathologies (Pennacchio et al., 1998; Franceschetti et al., 2007; Tegelberg et al., 2012). In addition to capturing region-specific alterations, we selected four time points from the pre-symptomatic stage (P14) to the advanced disease stage (6 months) to characterize the temporal patterns of molecular changes throughout disease progression in the *Cstb^−/−^* mouse brain.

When examining transcriptomic and proteomic alterations in different *Cstb^−/−^* brain regions and time points – first within each brain region separately and then across the regions – we observed the most striking changes in the cerebellum and cortex. Although transcriptomic analysis revealed widespread changes in the *Cstb^−/−^* brain, the proteomic alterations appeared more limited, possibly reflecting differences in analytical sensitivity. Notably, the cerebellum exhibited the most prominent changes in both datasets. The greatest overlap in DEGs during disease progression was observed between the cerebellum and cortex. Several inflammation and immune-response genes were consistently upregulated from P14 onwards, particularly in the cerebellum, with gene expression changes progressively extending to all brain regions. The observed upregulation of pro-inflammatory cytokine genes contributes to both inflammatory and anti-inflammatory processes and is known to recruit and activate immune cells, including microglia and peripheral immune cells, into the central nervous system (Kettenmann et al., 2011). Correspondingly, analysis of region-specific and shared KEGG pathways in the transcriptome revealed that immune response-related and lysosomal pathways, including the ‘phagosome’ pathway, were consistently enriched and upregulated across all brain regions during disease progression, suggesting the activation of phagocytic microglia. In line with this, morphological changes in activated microglia towards a phagocytic phenotype have previously been described in the brains of *Cstb^−/−^* mice, particularly in the early phases of disease progression (Tegelberg et al., 2012). Notably, unlike other epilepsy models in which hippocampal microglial activation is prominent (Bosco et al., 2018; Yu et al., 2023), immune-related gene expression was least pronounced in the *Cstb^−/−^* hippocampus. Despite the limited overlap between transcriptomic and proteomic KEGG pathways, the microglial activation pathway involving Janus kinase-signal transducer and activator of transcription (JAK-STAT) signalling was strongly upregulated in the cerebellum at 3 months in both datasets. As a critical mediator of cytokine signalling, the JAK-STAT pathway plays a central role in modulating immune responses (Nicolas et al., 2013). These findings align with our previous studies showing disrupted immune and defence response pathways in *Cstb^−/−^* microglia, including impaired interferon signalling, dysregulated JAK-STAT activity, cerebellar upregulation of inflammatory genes and early recruitment of peripheral immune cells (Joensuu et al., 2014; Okuneva et al., 2015; Körber et al., 2016). In addition, the upregulation of several glia-specific transcripts (Zamanian et al., 2012; Hasel et al., 2021) across brain regions supports our previous findings of early microgliosis and subsequent astrogliosis in the *Cstb^−/−^* mouse brain. Inflammatory signalling and immune system activation are frequently associated with neurodegenerative diseases (Mayne et al., 2020; Gumusgoz et al., 2024), and, based on these and our earlier data (Tegelberg et al., 2012; Okuneva et al., 2015, 2016), they also represent a hallmark feature of *Cstb*^−/−^ mice. In the periphery, *Cstb^−/−^* mice display elevated circulating cytokines and altered immune-cell populations, indicating early systemic immune activation alongside brain inflammation (Okuneva et al., 2016). Peripheral inflammation may amplify brain pathology by sustaining systemic cytokine signalling and increasing the susceptibility of glial cells to inflammatory activation, thereby contributing to disease progression.

The sustained elevation of inflammation-associated DEGs suggests prolonged microglial activation, promoting neurotoxicity, oxidative stress and chronic neuroinflammation that compromises neuronal resilience. In contrast to consistent upregulation of inflammatory processes, oxidative phosphorylation, the key energy metabolism process within mitochondria, was downregulated, particularly in *Cstb^−/−^* cerebellum from P14 onwards. In the cortex and hippocampus, the downregulation of oxidative phosphorylation becomes apparent later, at the clinical onset, indicating an increasing impact on the regulation of energy metabolism across different brain regions. Specifically, a large subset of mitochondrial genes encoding subunits of mitochondrial complex I in the electron transport chain was consistently downregulated in the cerebellum, suggesting impaired energy homeostasis and likely contributing to increased vulnerability of cerebellar neurons to oxidative stress and cell death due to CSTB deficiency (Lehtinen et al., 2009). Impaired electron transfer chain function is also implied by the reduced respiratory capacity in CSTB-deficient cerebellar synaptosomes (Gorski et al., 2023) and differentiating neural stem cells (Daura et al., 2021). Mitochondrial function plays a crucial role in cerebellar development, and deficits in the respiratory chain may impact both structural and functional processes in the brain (Lin et al., 2017; Anitha et al., 2023). The cerebellum's heightened susceptibility to neurodegeneration may be attributed to its high energy demands and the critical role of mitochondria in maintaining neuronal health, as granule cells, the primary excitatory neurons in the cerebellum, require exceptionally high energy to sustain synaptic activity and ensure proper circuit integrity (Clemente-Suárez et al., 2023; Gonzalez-Rodriguez and Marin-Valencia, 2024). Neurons rely predominantly on oxidative phosphorylation post-mitotically, whereas glycolysis is more prominent during developmental stages. However, impaired oxidative phosphorylation due to CSTB deficiency may trigger compensatory activation of alternative energy pathways and metabolic reprogramming – reverting to glycolysis – to meet cellular energy demands, reflecting an adaptive response to inflammation or oxidative stress in the *Cstb^−/−^* brain. In such cases, increased reliance on alternative energy substrates such as fatty acids, lactate and ketone bodies may be detected. Interestingly, altered expression of these alternative energy metabolism pathways was primarily observed in the *Cstb^−/−^* cerebellum transcriptome at later time points. Furthermore, metabolic reprogramming is a key driver in microglial immune response (Cheng et al., 2021), and metabolic dysregulation can exacerbate neuroinflammation, contributing to a self-perpetuating cycle in brain disease. This interplay between inflammation and mitochondrial dysfunction sustains neuronal damage, as chronic inflammation impairs mitochondrial function, while impaired mitochondrial function, in turn, fuels ongoing inflammation (Yang et al., 2021; Clemente-Suárez et al., 2023). Accordingly, abnormalities in brain energy metabolism and elevated lactate levels reflecting ongoing microglial activation have been observed in brains of patients with EPM1 (Hyppönen et al., 2023).

Lysosomes play a pivotal role in intracellular catabolism, autophagy, nutrient sensing and membrane repair (Lim and Zoncu, 2016), and their biogenesis and function are controlled by TFEB, which regulates the expression of the CLEAR network genes (Sardiello et al., 2009; Palmieri et al., 2011; Settembre et al., 2011). Under stress, lysosome-localized mTORC1 inactivation leads to TFEB dephosphorylation and nuclear translocation, where it regulates CLEAR network transcription (Roczniak-Ferguson et al., 2012; Doronzo et al., 2021). Transcriptome analysis revealed consistent upregulation of the ‘lysosome’ pathway across most *Cstb^−/−^* brain regions and time points, further supported by the integrated transcriptomic and proteomic data from the cortex at 3 months. In addition, lysosomal DEGs and CLEAR network genes also showed expression changes. These findings may indicate an upregulated lysosomal demand to sustain cellular clearance or homeostasis. Notably, multiple genes encoding vacuolar type-H^+^-ATPase subunits were downregulated in *Cstb^−/−^* cerebellum at 3 and 6 months, while genes encoding lysosomal hydrolases were upregulated, particularly in the cerebellum and cerebral cortex from 1 month onwards. The V-ATPase is crucial for maintaining the acidic pH needed for cathepsin and other hydrolase functions (Collins and Forgac, 2020). The downregulation of V-ATPase subunit genes suggests a reduction in lysosomal acidification capacity, which may lead to cellular stress and accumulation of cellular waste in lysosomes. This may further result in lysosomal membrane rupture, which leads to the leakage of hydrolases into the cytoplasm, potentially damaging cell organelles and resulting in cell death (Wang et al., 2018; Collins and Forgac, 2020). The upregulation of lysosomal hydrolases, such as cathepsins and hexosaminidases, may also indicate cells' attempts to compensate for lysosomal leakage. CSTB inhibits cysteine cathepsins (Bode et al., 1988; Stubbs et al., 1990; Čeru et al., 2010) and protects against proteolytic damage. Its absence may increase cathepsin activity, promoting lysosomal membrane permeabilization, cytosolic leakage and activation of proinflammatory pathways. In chronic inflammatory states, the activated microglia may release excessive neurotoxic molecules, including lysosomal cathepsins (Pišlar et al., 2021). This can trigger further activation of the inflammatory microglial phenotype. Leakage of lysosomal cathepsins can disrupt mitochondrial metabolism and promote inflammation, as shown in macrophages (Bussi et al., 2022). While transient lysosomal protease leakage occurs during endomembrane transport and repair, it may contribute to mitochondrial dysfunction when CSTB is absent. Our findings further suggest that progressive lysosomal dysregulation contributes to the exacerbation of neuroinflammation in the *Cstb^−/−^* mouse brain.

In addition to the consistent upregulation of immune-related pathways and downregulation of oxidative phosphorylation in the cerebellum, further analysis of integrated transcriptomic and proteomic pathways revealed a significant upregulation of ‘apoptosis’ in the *Cstb^−/−^* cerebellum at 3 months, suggesting increased neuronal cell death through apoptosis. This finding is not unexpected, given that the cerebellum of CSTB-deficient mice exhibits the most severe pathological alterations, including extensive apoptotic death of cerebellar granule cells, beginning at 1 month of age (Pennacchio et al., 1998) and leading to nearly 50% loss of the cerebellar volume by 6 months of age (Tegelberg et al., 2012; Manninen et al., 2013, 2014). The integrated data from the cortex at 3 months revealed a marked decrease in abundance of G-protein beta subunits across multiple synaptic signalling pathways, vital for maintaining cognitive and neuronal function (Wong et al., 2023). Reduced G-protein abundance may contribute to impaired G-protein-coupled receptor (GPRC) neuronal signalling and neurotransmitter responses (Betke et al., 2012; Tennakoon et al., 2021) underlying neurodegenerative processes in the *Cstb^−/−^* cortex. Furthermore, the upregulation of the nuclear receptor PPAR signalling pathway may reflect metabolic reprogramming in lipid metabolism and modulation of mitochondrial function and inflammatory responses (Zolezzi et al., 2017).

Among childhood-onset neurodegenerative disorders, the group of neuronal ceroid lipofuscinoses (NCLs) are collectively the most frequent inherited neurodegenerative diseases in children. Their clinical presentations differ from EPM1 in being rapidly progressing, leading typically to early death (Ziółkowska et al., 2025). Despite this, they share common pathogenetic mechanisms with EPM1, including neuroinflammatory and neuronal alterations, oxidative stress, dysregulation of lysosomal pathways and impaired mitochondrial function (Simonati and Williams, 2022; Ziółkowska et al., 2025). Transcriptomic and proteomic analyses have been carried out in brains of NCL mouse models allowing comparison to our data. Transcriptomic analyses in the model for infantile-onset NCL, CLN1, in which early neuropathological and inflammatory changes particularly affect thalamus (Kielar et al., 2007), showed relatively few gene expression changes at the pre-symptomatic stage (Elshatory et al., 2003) but a marked upregulation of inflammatory and immune-related genes during symptomatic and late stages (Jalanko et al., 2005; Qiao et al., 2007). At the proteomic level, early changes involved neuronal processes and increased abundances of tricarboxylic acid cycle and mitochondrial proteins, followed at later stages by decreased levels of mitochondrial proteins and a pronounced reduction in myelin-associated proteins in the thalamus (Tikka et al., 2016). In the cortex, early stages were characterized by changes primarily affecting metabolic, mitochondrial and autophagy-related pathways, while inflammatory and immune responses were less prominent than in other affected regions (Nelvagal et al., 2020).

In the mouse model for the late infantile-onset CLN2 disease, inflammation becomes evident typically early symptomatic stage, with microglia and astrocyte activation occurring earlier in the cerebellum than in other brain regions (Domowicz et al., 2021). Transcriptomic changes are modest at pre-symptomatic stages, with more pronounced alterations in the cerebellum as disease progresses (Domowicz et al., 2019, 2021). At the symptomatic stages, there is a significant dysregulation of lysosomal, phagolysosomal and autophagy-related pathways, along with downregulation of neuronal and synaptic functions (Domowicz et al., 2021). Transcriptomic and proteomic analyses in a juvenile-onset CLN3 mouse model indicate that the early disease stages are characterized by, similarly to the *Cstb^−/−^* mouse model, immune and inflammatory changes in the cerebellum, accompanied by impaired lysosomal function that may compromise microglial neuroprotective roles and promote neuroinflammation (Brooks et al., 2003; Yasa et al., 2024).

Taken together, these findings support the view that neuroimmune activation, mitochondrial dysfunction and lysosome-related pathways represent core molecular features across many childhood-onset neurodegenerative disorders. They frequently exhibit upregulation of lysosomal genes, likely reflecting a compensatory response to neuroinflammation and phagocytic demand. Interestingly, in the *Cstb^−/−^* mouse model, several lysosomal genes encoding vacuolar type-H^+^-ATPase subunits are downregulated from disease onset onwards, suggesting impaired lysosomal acidification. Defects in lysosomal acidification and membrane integrity have been linked to disrupted autophagy–lysosome function in microglia, potentially amplifying cytokine release, persistent neuroinflammation and neurodegeneration (Quick et al., 2023).

In the *Cstb^−/−^* mouse brains, proteomic analyses revealed considerably fewer DAPs than the DEGs, and only a small number of these proteins remained significant after multiple-testing correction, likely reflecting the limited sensitivity of proteomic approaches (Timp and Timp, 2020). Therefore, we also examined proteins with unadjusted *P*-values to obtain a broader view of potential proteomic changes. These unadjusted findings highlight trends in protein abundance and offer preliminary evidence that may inform future targeted validation. The analyses revealed the increased abundance of five proteins – CLU, APOE, PRDX6, CTSD and ALDOC – across all three brain regions, suggesting their potential as biomarkers for disease progression (Letronne et al., 2016; Pankiewicz et al., 2020; Wang et al., 2023; Mares et al., 2025). APOE and CLU are involved in lipid transport and metabolism, neuronal plasticity and cellular stress responses, and both are expressed by astrocytes and microglia in the central nervous system. They are also known to play roles in modulating inflammatory response (Yeh et al., 2016; Gross et al., 2023; Windham and Cohen, 2024). Given these roles and broad cellular expression pattern, APOE may contribute to the progression of EPM1 through various parallel pathways. From 1 month onwards, APOE abundance increases, particularly in the *Cstb^−/−^* cerebellum, concurrent with strong microglial activation and upregulation of inflammatory genes. This elevation may further drive microglia toward a more reactive, pro-inflammatory phenotype, potentially modulating both their activation states and phagocytic capacity during disease progression. Moreover, because APOE is a central regulator of lipid transport in the brain, alterations in lipid metabolism can affect cellular membrane composition, which may contribute to neuronal damage and neuroinflammation during EPM1 disease progression. Furthermore, increased abundance of APOE suggests that enhanced lipid metabolism may be required to meet the metabolic demands of protective cellular functions, such as phagocytosis (Loving and Bruce, 2020). Finally, alterations in APOE expression have also been associated with impaired brain energy metabolism, characterized by reduced mitochondrial oxidative phosphorylation and a shift toward aerobic glycolysis (Budny et al., 2025). Notably, our data suggest that these energy metabolism-related processes appear to be disrupted in the *Cstb^−/−^* cerebellum from P14 onwards.

PRDX6 is mainly an astrocytic multifunctional enzyme that combines peroxidase activity with antioxidant acidic calcium-independent phospholipase A2 (aiPLA2) activity, as well as lysophosphatidylcholine acyltransferase (LPCAT) activity. It has previously been associated with oxidative stress, phospholipid homeostasis and redox balance in central nervous system diseases, either exacerbating or attenuating neuronal damage (Xue et al., 2024). In line with these functions, PRDX6 abundance increases markedly in the *Cstb^−/−^* brain from 1 month of age onwards, particularly in the cerebellum. Notably, this upregulation temporally coincides with the onset of myoclonus, astrogliosis and elevated microglial proinflammatory cytokine expression (Tegelberg et al., 2012; Okuneva et al., 2015; Pollari et al., 2023), suggesting ongoing activation of inflammatory pathways and heightened oxidative stress potentially associated with increased PRDX6 abundance. Thus, PRDX6 may play a dual role in EPM1 pathology by limiting oxidative damage via its peroxidase activity, while excessive aiPLA2 activity may exacerbate neuroinflammation. Accordingly, increased PRDX6 abundance may reflect an inflammation-associated antioxidant response, in which PRDX6 is increased together with other antioxidant enzymes. Nonetheless, this association highlights PRDX6 as a potential therapeutic target, particularly in the context of inflammation-driven redox imbalance, warranting further investigation into its role in EPM1. CTSD, a lysosomal enzyme, and ALDOC, a glycolytic enzyme, further underscore the involvement of lysosomal and metabolic pathways. Based on the Human Protein Atlas secretome (Uhlén et al., 2019), APOE and CLU can be identified in human blood, highlighting their potential as biomarkers in preclinical and clinical settings.

In conclusion, our findings underscore the complexity of disease progression in the *Cstb^−/−^* mouse brain and underscore differential immune responses, mitochondrial function and lysosomal homeostasis as key mechanisms, with their impact increasing as the disease progresses. Furthermore, the cerebellum shows the earliest and most pronounced alterations, highlighting its central role in disease pathophysiology. These findings provide not only insights into the molecular basis of the disease mechanisms but also into potential biomarkers and therapeutic targets.

## MATERIALS AND METHODS

### Mouse model

The CSTB-deficient (*Cstb^−/−^*) mouse strain used in this study is 129S2/SvHsd5-*Cstb^tm1Rm^*, derived from The Jackson Laboratory strain 129-*Cstb^tm1Rm/J^* (stock no. 003486) (Pennacchio et al., 1998). Age-matched wt mice of the 129S2/SvHsd5 were used as controls. All mice used were male. The research protocols were approved by the Animal Ethics Committee of the State Provincial Office of Southern Finland (decisions ESAVI/10765/04.10.07/2015, ESAVI/471/2019 and ESAVI/2242/2022).

### Tissue dissection

For omics analysis, adult mice aged 1, 3 and 6 months were subjected to a 12 h fasting period, followed by a 2-4 h feeding period before tissue collection. P14 mice were kept with their mother until immediately prior to euthanasia. Mice were euthanized using CO_2_, and the brain was extracted, bisected along the midline and dissected into the cerebellum, the cerebral cortex and the hippocampus using brushes. The cerebral cortex was then divided into three equal sections, of which the middle section [approximately Bregma −1.22 mm to 1.10 mm, as defined by Paxinos and Franklin (2001)] was used for RNA sequencing and the front section (approximately Bregma 1.10 mm to 3.56 mm) was used for liquid chromatography–tandem mass spectrometry (LC/MS-MS) proteomics. All tissue samples were immediately snap frozen in liquid nitrogen and stored at −80°C.

For RNA sequencing, total RNA was collected from the brain (cerebellum, hippocampus, cortex) at four time points: P14, 1 month, 3 months (*n*=8 mice per genotype and age) and 6 months (*n*=9 for wt mice and *n*=7 for *Cstb^−/−^* mice).

For LC/MS-MS proteomics, proteins were extracted from the brain (cerebellum, hippocampus, cortex) of male mice at four time points: P14, 1 month, 3 months (*n*=8 mice per genotype and age) and 6 months (*n*=6 mice per genotype for cerebellar and hippocampal samples, and *n*=9 for wt and *n*=7 for *Cstb^−/−^* mouse cerebral cortex samples).

### RNA isolation

For RNA isolation from brain tissue, the frozen tissue samples were transferred to Lysing Matrix D tubes (MP Biomedicals) with 600 µl RLT Plus (Qiagen) containing 2-mercaptoethanol (Acros Organics) and homogenized for 3×20 s in a SpeedMill PLUS bead homogenizer (Analytic Jena) with a pre-cooled rotor (−80°C). RNA was isolated using an RNeasy Plus Mini (Qiagen) kit in accordance with the manufacturer's instructions and eluted in 50 µl RNase-free water. RNA concentrations were measured using a Denovix Ds11 spectrophotometer/fluorometer (GC Biotech), while RNA integrity and quality were analysed using Tapestation (Agilent) and a Qubit HS RNA (Thermo Fisher Scientific).

### RNA sequencing

Bulk RNA sequencing was performed in the Biomedicum Functional Genomics Unit (University of Helsinki, Helsinki, Finland) as previously described in Kang et al. (2024). Methods were based on the Drop-seq method for multiplexing (Macosko et al., 2015; Freitag et al., 2020). Briefly, sequencing was performed on the Illumina NextSeq 500 system in two runs using NextSeq High Output 75 cycle flow cell-sequencing chips. Fastq files were generated (Bcl2fastq, Illumina), data quality was evaluated (FastQC), and results were summarized (MultiQC). Reads were tagged with sample-specific barcodes and unique molecular identifiers (UMIs). Tagged reads were then trimmed for 5′ adapters and 3′ poly A tails, and poorly aligned reads were further processed using Trimmomatic (Bolger et al., 2014). The data were annotated using GENCODE mouse M24 (GRCm38.p6) (Frankish et al., 2019) and processed using Drop-seq tools. The completed aligned data was separated into gene expression matrices based on sample identity.

### Protein isolation for LC/MS-MS

For LC-MS/MS, frozen tissue samples between 20 and 70 mg were weighed and transferred to Lysing Matrix D tubes (MP Biomedicals) containing 600-900 µl Trizol^TM^ Reagent (Thermo Fisher Scientific), with the volume adjusted according to tissue weight. Tissues were homogenized using a SpeedMill PLUS homogenizer. Phase separation was performed by adding chloroform (Sigma-Aldrich) at a ratio of 0.2 ml per 1 ml Trizol^TM^ Reagent used, followed by centrifugation for 15 min at 12,000 ***g*** at 4°C. Proteins were precipitated from the organic phase according to the manufacturer's instructions. Pellets were resuspended in 200 µl of 50 mM ammonium bicarbonate/6 M urea/1× Pierce protease and phosphatase inhibitor mini tablets (Thermo Fisher Scientific) and boiled at 95°C for 5 min, centrifuged for 5 min at 10,000 ***g*** at 4°C, aliquoted and stored at −20°C.

### Protein digestion, LC-MS/MS and data analysis

Protein concentration was estimated using a BCA assay (Pierce), and equal amounts of protein samples were aggregated on amine beads (Batth et al., 2019). For on-bead digestion, 50 mM ammonium bicarbonate buffer was added to beads. Proteins were reduced with 10 mM DTT for 30 min at 37°C and alkylated with 20 mM iodoacetamide for 30 min at room temperature in the dark. Subsequently, 0.5 µg trypsin was added, and digestion was carried out overnight at 37°C. The beads were separated using a magnet, and the supernatant was transferred to a new tube and acidified. The tryptic peptides were desalted using C18 StageTips before mass spectrometry analysis.

LC-MS/MS analysis of the resulting peptides was conducted using an Evosep liquid chromatography system coupled to a QExactive HF Hybrid Quadrupole-Orbitrap mass spectrometer (Thermo Electron) with a nanoelectrospray ion source (EasySpray, Thermo Electron). Peptides were separated using an extended method involving an 88-min gradient from 2% to 30% (v/v) acetonitrile in 0.1% (v/v) formic acid, followed by column washing with 90% (v/v) acetonitrile in 0.1% (v/v) formic acid for 20 min at a flow rate of 0.3 μl/min. All LC-MS/MS analyses were performed in data-dependent acquisition mode, wherein the most intense peptides were automatically selected for fragmentation via high-energy collision-induced dissociation.

Raw files from LC-MS/MS analyses were submitted to MaxQuant 1.6.17.0 software (Cox and Mann, 2008) for peptide/protein identification. Parameters were set as follows: carbamidomethyl (C) was set as a fixed modification, while protein N-acetylation and methionine oxidation were set as variable modifications. The first search had an error window of 20 ppm and the main search error of 6 ppm. Trypsin without the proline restriction enzyme option was used, with up to two missed cleavages allowed. A minimum of one unique peptide was required for protein identification, and a false discovery rate (FDR) of 0.01 (1%) was applied at both the peptide and protein levels. The UniProt mouse database was used, and reversed sequences were generated to estimate FDR rates.

MaxQuant output files were loaded into the Perseus 1.6.1.3 software (Tyanova et al., 2016) for further data filtering and statistical analysis. Identifications from potential contaminants and reversed sequences were removed, and normalized intensities (LFQ) were transformed to a log10 scale. The dataset was filtered to retain only proteins with at least 50% valid values in at least one experimental group.

### Computational analysis

For differential expression analysis, low-expressed genes were filtered out, and the edgeR R package pipeline was utilized (Robinson et al., 2010; McCarthy et al., 2012). The analysis compared *Cstb^−/−^* and wt mice across each brain region and time point. Quasi-likelihood negative binomial generalized log-linear models were employed to fit the count data, and empirical Bayes quasi-likelihood F-tests were applied to obtain gene-specific and adjusted *P*-values (Robinson and Oshlack, 2010; Lund et al., 2012). The adjusted *P*-value threshold was established at 0.05 to maintain a 5% false-positive rate for significant genes.

The differential abundance of proteins analysis was conducted similarly to the differential expression analysis, utilizing limma R package for linear model fitting and empirical Bayes moderation (Smyth, 2004). Both gene and protein EGSEAs were conducted using the EGSEA R package (Alhamdoosh et al., 2017), with an adjusted *P*-value threshold of 0.05 to identify significant pathways. The KEGG and GO collections were used for pathway categorization.

Illustrations for figures were produced using base functions from RStudio, R packages ggplot2 (Wickham, 2016), pheatmap R package version 1.0.12 and Adobe Illustrator 2023-2025.

## Supplementary Material

10.1242/dmm.052681_sup1 Supplementary information

Table S1. Differentially expressed genes (DEGs) identified in the cerebellum (CB), cerebral cortex (CTX), and hippocampus (HC) of *Cstb^-/-^* (KO) vs. wild-type (WT) mice at time points (P14, 1, 3, 6 months).

Table S2. Differentially regulated Kyoto Encyclopedia of Genes and Genomes (KEGG) pathways identified in cerebellum (CB), cerebral cortex (CTX), and hippocampus (HC) of *Cstb^-/-^* (KO) vs. wildtype (WT) mice across time points (P14, 1, 3, 6 months).

Table S3. Shared, enriched Kyoto Encyclopedia of Genes and Genomes (KEGG) pathways and overlapping pathway-associated genes across cerebellum (CB), cerebral cortex (CTX) and hippocampus (HC) of *Cstb^-/-^* (KO) vs. wild-type (WT) mice across time points (P14, 1, 3, 6 months).

Table S4. Genes linked to the immune system and inflammation response; extracted from associated GO pathways (GO:0007250, GO:0008009, GO:0006954, GO:0045087, GO:0006955, GO:0051607, GO:0007249, GO:0008063, GO:0032735, GO:0051092, GO:0002224, GO:0032722, GO:0002526, GO:0007252).

Table S5. Differentially expressed genes (DEGs) linked to immune and inflammatory Gene Ontology (GO) gene sets in cerebellum (CB), cerebral cortex (CTX), hippocampus (HC) across time points (P14, 1, 3, 6 months) of *Cstb^-/-^* (KO) vs. wild-type (WT) mice.

Table S6. Differentially regulated Gene Ontology (GO) pathways identified in the cerebellum (CB), cerebral cortex (CTX), and hippocampus (HC) of *Cstb^-/-^* (KO) vs. wild-type (WT) mice across time points (P14, 1, 3, 6 months).

Table S7. Differential expression of MitoCarta-annotated mitochondrial genes detected in cerebellum (CB), cerebral cortex (CTX), and hippocampus (HC) of *Cstb^-/-^* (KO) vs. wild-type (WT) mice across time points (P14, 1, 3, 6 months).

Table S8. Genes linked to the lysosomal cellular compartment; extracted from Gene Ontology (GO) lysosome pathway (GO:0005764).

Table S9. Differentially expressed genes (DEGs) linked to Gene Ontology (GO) lysosome gene set in the cerebellum (CB), cerebral cortex (CTX), hippocampus (HC) across time points (P14, 1, 3, 6 months) of *Cstb^-/-^* (KO) vs.wild-type (WT) mice.

Table S10. Differentially abundant proteins (DAPs) identified in the cerebellum (CB), cerebral cortex (CTX), and hippocampus (HC) of *Cstb^-/-^* (KO) vs. wild-type (WT) mice at different time points (P14, 1, 3, 6 months).

Table S11. The Kyoto Encyclopedia of Genes and Genomes (KEGG) pathways associated with differentially abundant proteins in the cerebellum (CB), cerebral cortex (CTX), and hippocampus (HC) of *Cstb^-/-^* (KO) vs. wild-type (WT) mice at different time points (P14, 1, 3, 6 months).

Table S12. Shared Kyoto Encyclopedia of Genes and Genomes (KEGG) pathways between transcriptomic (DEG-based) and proteomic (DAP-based) pathway analyses in cerebellum (CB), cerebral cortex (CTX), and hippocampus (HC) of *Cstb^-/-^* (KO) vs. wild-type (WT) mice across time points (P14, 1, 3, 6 months).

Table S13. Overlapping differentially expressed genes (DEGs) and differentially abundant proteins (DAPs) identified in the cerebellum (CB), cerebral cortex (CTX), and hippocampus (HC) of *Cstb^-/-^* (KO) vs. wild-type (WT) mice across time points (P14, 1, 3, and 6 months).
