# Supplementary material for: Transcriptomic and proteomic insights into progressive myoclonus epilepsy type 1
Source: Dis Model Mech. 2026 May 5;19(4):dmm052681. doi: 10.1242/dmm.052681 (PMC13225231; doi:10.1242/dmm.052681)
Supplement: Supplementary information [file dmm-19-052681-s1.pdf]

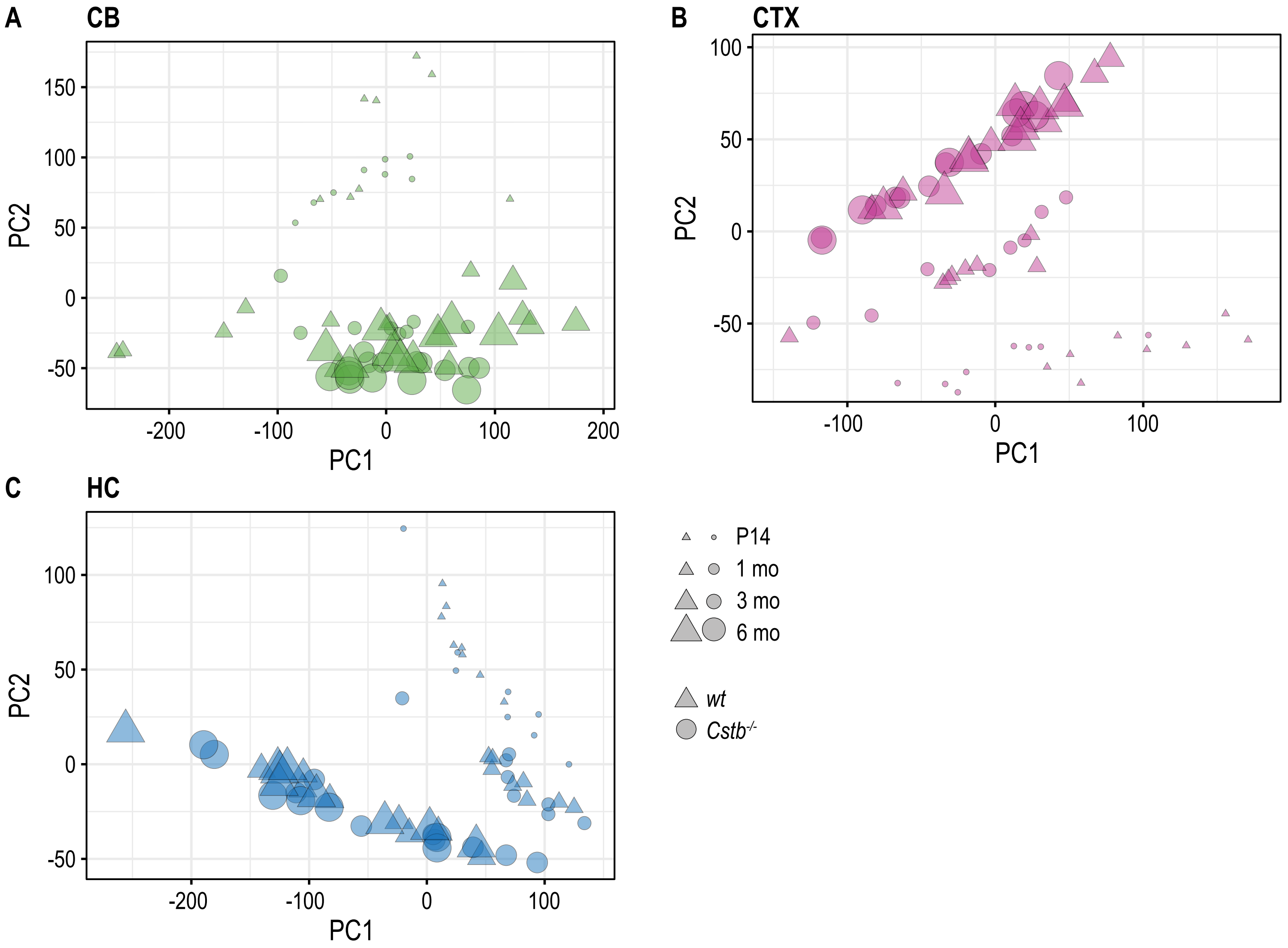

Fig. S1. Principal component analysis of RNA sequencing data across *Cstb$^{-/-}$* mouse brain regions and time points A-C Principal component analysis (PCA) plot showing the distribution of RNA sequencing data from *Cstb$^{-/-}$* and wild type (*wt*) mice in **A**) cerebellum, **B**) cortex, **C**) hippocampus across genotypes and time points. Principal component 1 (PC1) and principal component 2 (PC2) are displayed.

Abbreviations: CB, cerebellum; CTX, cortex; HC, hippocampus.

Disease Models & Mechanisms • Supplementary information

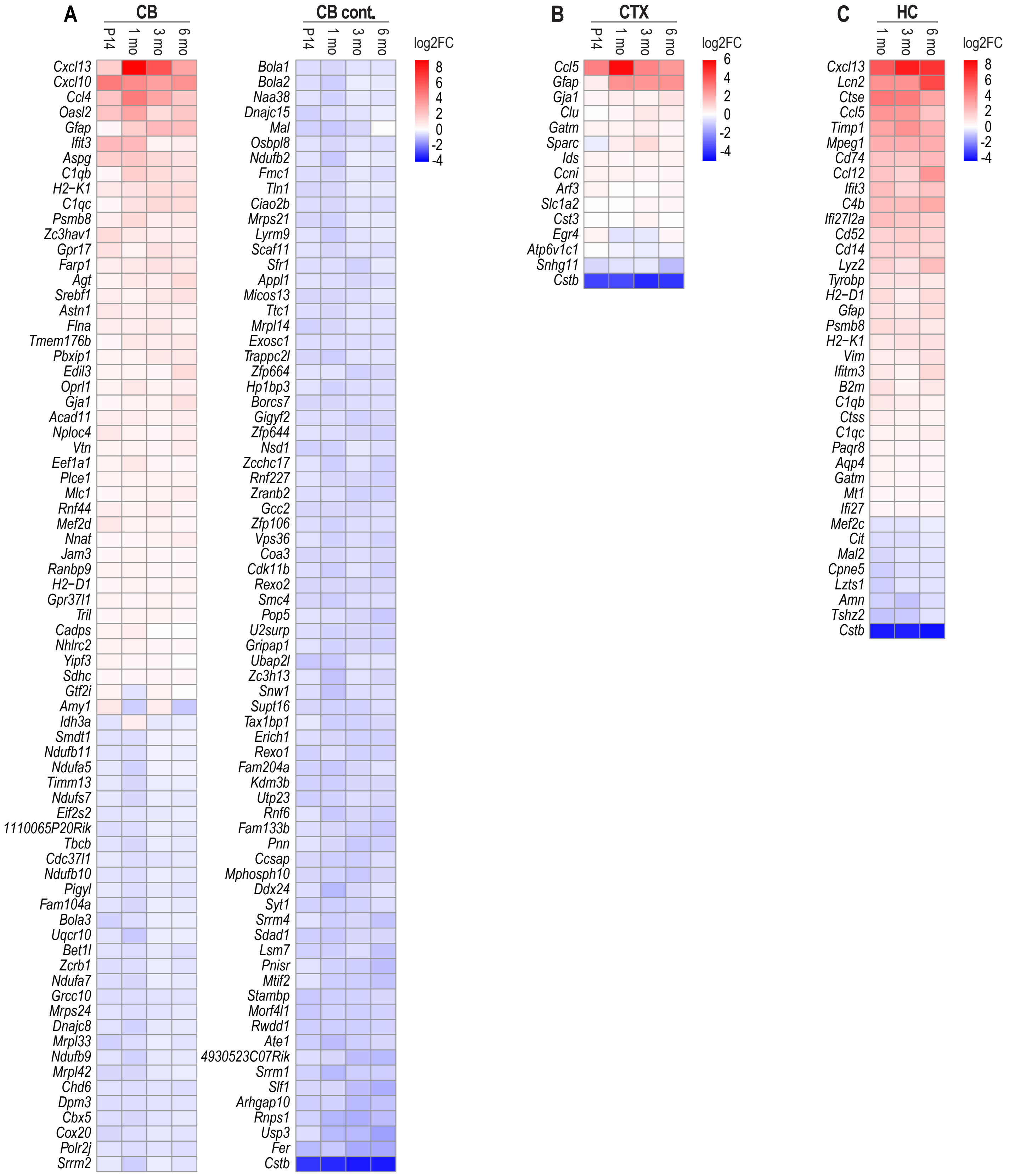

Fig. S2. Heatmaps of differentially expressed genes in the *Cstb*$^{-/-}$ mouse brain

A-C Heatmaps illustrating **A**) Cerebellum: the 146 significant DEGs from P14 to 6 months; **B**) Cortex: the 15 significant DEGs from P14 to 6 months. **C**) Hippocampus: the 38 significant DEGs, all showing consistent differential expression from 1 to 6 months, including *Cstb*. The colour scale represents log2-fold changes (log2FC) in gene expression, with red indicating upregulation and blue downregulation.

Abbreviations: CB, cerebellum; CTX, cortex; HC, hippocampus.

Disease Models & Mechanisms • Supplementary information

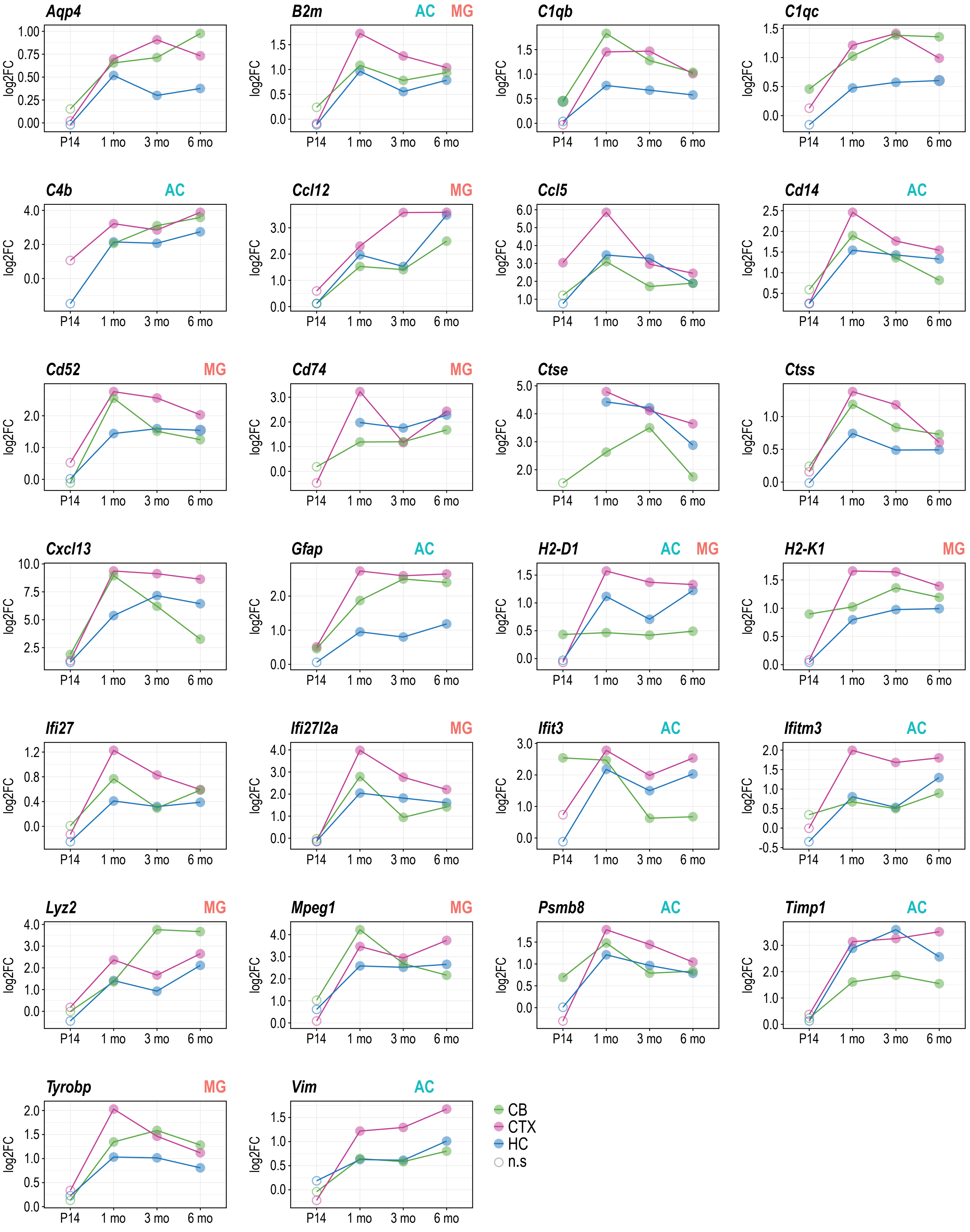

Fig. S3. Differentially expressed genes with consistent directional changes during disease progression in the *Cstb*$^{-/-}$ mouse brain

Line plots of 26 genes displayed based on their differential expression at a minimum of three consecutive time points, with consistent up- or downregulation. The Y-axis represents log2-fold changes (log2FC). Filled circles indicate significant values (p-adj < 0.05), while unfilled circles indicate non-significant values (p-adj > 0.05).

Abbreviations: AC, astrocyte marker; MG, microglia marker, n.s, non-significant.

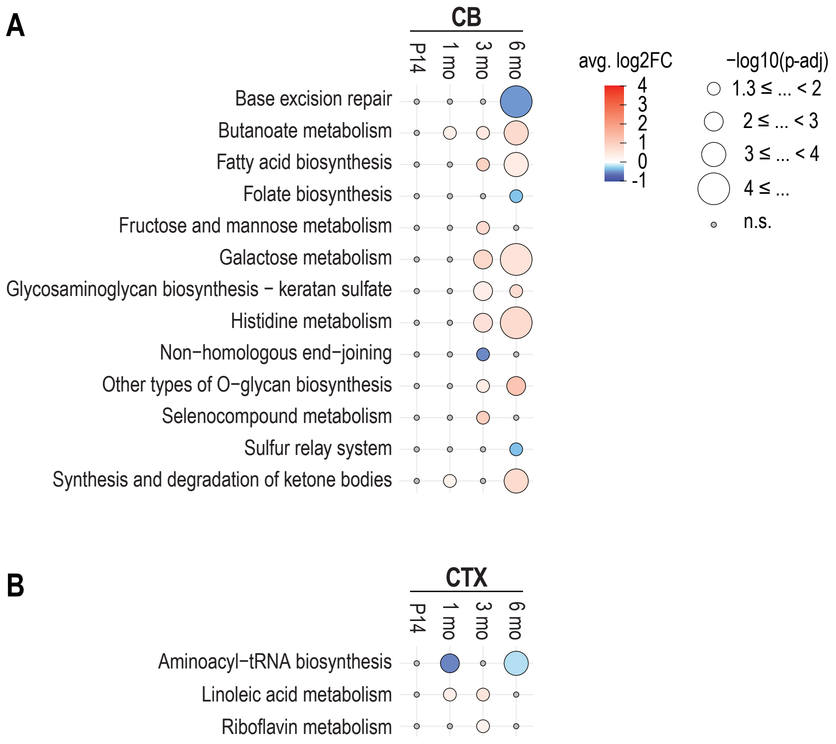

Fig. S4. Pathways enriched exclusively in the cerebellum and cortex, identified through the transcriptome EGSEA analysis of the *Cstb*$^{-/-}$ mouse brain

A-B Dot plots showing **A)** 13 pathways exclusively represented in the cerebellum and **B)** three pathways in the cortex during disease progression. Circle size indicates statistical significance as -log10(p-adj), while colour intensity denotes the average directional log2-fold change (log2FC), with blue representing downregulation and red representing upregulation. Abbreviations: CB, cerebellum; CTX, cortex; n.s., non-significant with p-adj > 0.05.

Disease Models & Mechanisms • Supplementary information

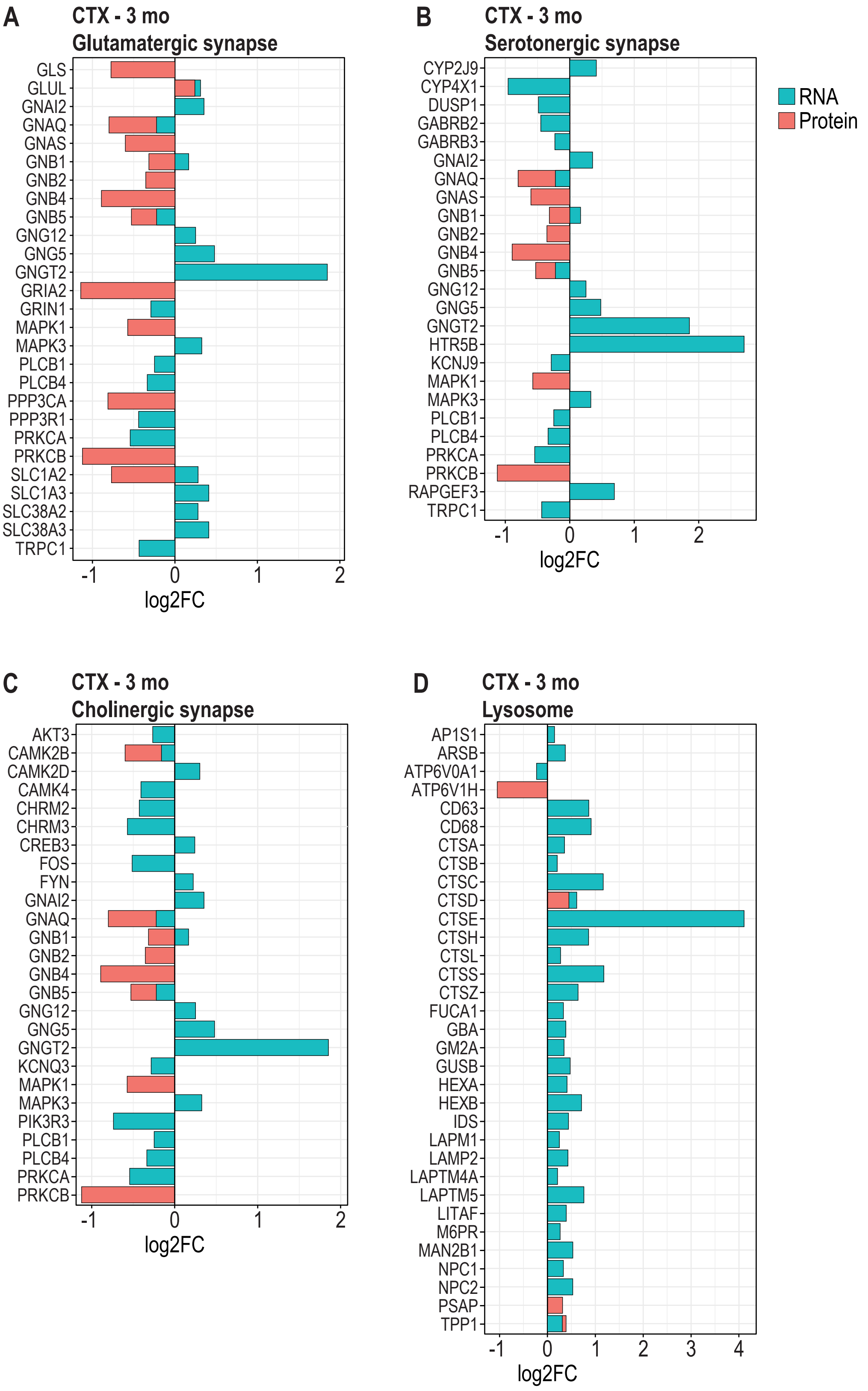

Fig. S5. Comparative EGSEA analysis of the proteome and transcriptome in the cortex of *Cstb*$^{-/-}$ mouse, highlighting synapse- and lysosome-related genes and proteins at 3 months

A-D Bar charts depicting KEGG pathway annotated genes (teal) and proteins (orange) enriched in the cortex at 3 months related to **A**) glutamatergic synapse; **B**) serotonergic synapse; **C**) cholinergic synapse; **D**) lysosome pathways. The X-axis represents directional log2-fold changes (log2FC).

Disease Models & Mechanisms • Supplementary information

Table S1. Differentially expressed genes (DEGs) identified in the cerebellum (CB), cerebral cortex (CTX), and hippocampus (HC) of *Cstb*$^{-/-}$ (KO) vs. wild-type (WT) mice at time points (P14, 1, 3, 6 months).

Available for download at
https://journals.biologists.com/dmm/article-lookup/doi/10.1242/dmm.052681#supplementary-data

Table S2. Differentially regulated Kyoto Encyclopedia of Genes and Genomes (KEGG) pathways identified in cerebellum (CB), cerebral cortex (CTX), and hippocampus (HC) of *Cstb*$^{-/-}$ (KO) vs. wild-type (WT) mice across time points (P14, 1, 3, 6 months).

Available for download at
https://journals.biologists.com/dmm/article-lookup/doi/10.1242/dmm.052681#supplementary-data

Table S3. Shared, enriched Kyoto Encyclopedia of Genes and Genomes (KEGG) pathways and overlapping pathway-associated genes across cerebellum (CB), cerebral cortex (CTX) and hippocampus (HC) of *Cstb*$^{-/-}$ (KO) vs. wild-type (WT) mice across time points (P14, 1, 3, 6 months).

Available for download at
https://journals.biologists.com/dmm/article-lookup/doi/10.1242/dmm.052681#supplementary-data

Table S4. Genes linked to the immune system and inflammation response; extracted from associated GO pathways (GO:0007250, GO:0008009, GO:0006954, GO:0045087, GO:0006955, GO:0051607, GO:0007249, GO:0008063, GO:0032735, GO:0051092, GO:0002224, GO:0032722, GO:0002526, GO:0007252).

Available for download at
https://journals.biologists.com/dmm/article-lookup/doi/10.1242/dmm.052681#supplementary-data

Table S5. Differentially expressed genes (DEGs) linked to immune and inflammatory Gene Ontology (GO) gene sets in cerebellum (CB), cerebral cortex (CTX), hippocampus (HC) across time points (P14, 1, 3, 6 months) of *Cstb*$^{-/-}$ (KO) vs. wild-type (WT) mice.

Available for download at
https://journals.biologists.com/dmm/article-lookup/doi/10.1242/dmm.052681#supplementary-data

Table S6. Differentially regulated Gene Ontology (GO) pathways identified in the cerebellum (CB), cerebral cortex (CTX), and hippocampus (HC) of *Cstb*$^{-/-}$ (KO) vs. wild-type (WT) mice across time points (P14, 1, 3, 6 months).

Available for download at
https://journals.biologists.com/dmm/article-lookup/doi/10.1242/dmm.052681#supplementary-data

Table S7. Differential expression of MitoCarta-annotated mitochondrial genes detected in cerebellum (CB), cerebral cortex (CTX), and hippocampus (HC) of *Cstb*$^{-/-}$ (KO) vs. wild-type (WT) mice across time points (P14, 1, 3, 6 months).

Available for download at
https://journals.biologists.com/dmm/article-lookup/doi/10.1242/dmm.052681#supplementary-data

Table S8. Genes linked to the lysosomal cellular compartment; extracted from Gene Ontology (GO) lysosome pathway (GO:0005764).

Available for download at
https://journals.biologists.com/dmm/article-lookup/doi/10.1242/dmm.052681#supplementary-data

Table S9. Differentially expressed genes (DEGs) linked to Gene Ontology (GO) lysosome gene set in the cerebellum (CB), cerebral cortex (CTX), hippocampus (HC) across time points (P14, 1, 3, 6 months) of *Cstb*$^{-/-}$ (KO) vs.wild-type (WT) mice.

Available for download at
https://journals.biologists.com/dmm/article-lookup/doi/10.1242/dmm.052681#supplementary-data

Table S10. Differentially abundant proteins (DAPs) identified in the cerebellum (CB), cerebral cortex (CTX), and hippocampus (HC) of *Cstb*$^{-/-}$ (KO) vs. wild-type (WT) mice at different time points (P14, 1, 3, 6 months).

Available for download at
https://journals.biologists.com/dmm/article-lookup/doi/10.1242/dmm.052681#supplementary-data

Table S11. The Kyoto Encyclopedia of Genes and Genomes (KEGG) pathways associated with differentially abundant proteins in the cerebellum (CB), cerebral cortex (CTX), and hippocampus (HC) of *Cstb*$^{-/-}$ (KO) vs. wild-type (WT) mice at different time points (P14, 1, 3, 6 months).

Available for download at
https://journals.biologists.com/dmm/article-lookup/doi/10.1242/dmm.052681#supplementary-data

Table S12. Shared Kyoto Encyclopedia of Genes and Genomes (KEGG) pathways between transcriptomic (DEG-based) and proteomic (DAP-based) pathway analyses in cerebellum (CB), cerebral cortex (CTX), and hippocampus (HC) of *Cstb*$^{-/-}$ (KO) vs. wild-type (WT) mice across time points (P14, 1, 3, 6 months).

Available for download at
https://journals.biologists.com/dmm/article-lookup/doi/10.1242/dmm.052681#supplementary-data

Table S13. Overlapping differentially expressed genes (DEGs) and differentially abundant proteins (DAPs) identified in the cerebellum (CB), cerebral cortex (CTX), and hippocampus (HC) of *Cstb*$^{-/-}$ (KO) vs. wild-type (WT) mice across time points (P14, 1, 3, and 6 months).

Available for download at
https://journals.biologists.com/dmm/article-lookup/doi/10.1242/dmm.052681#supplementary-data
